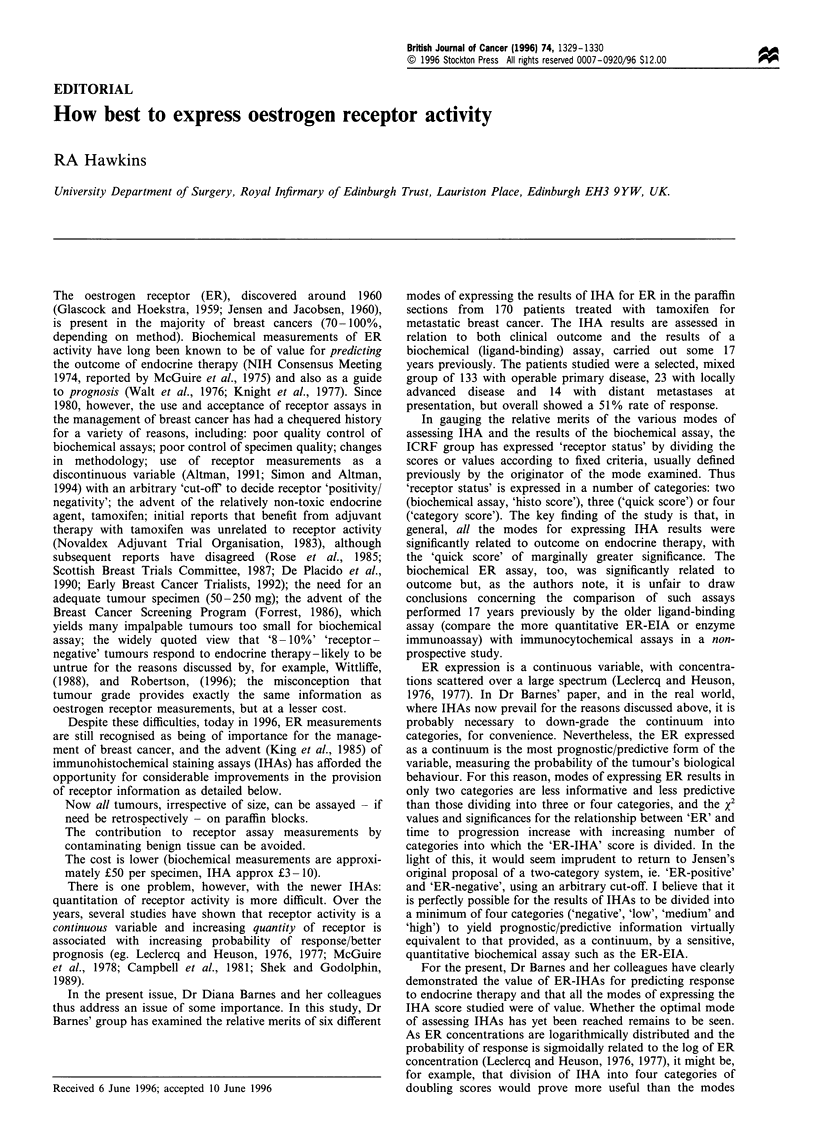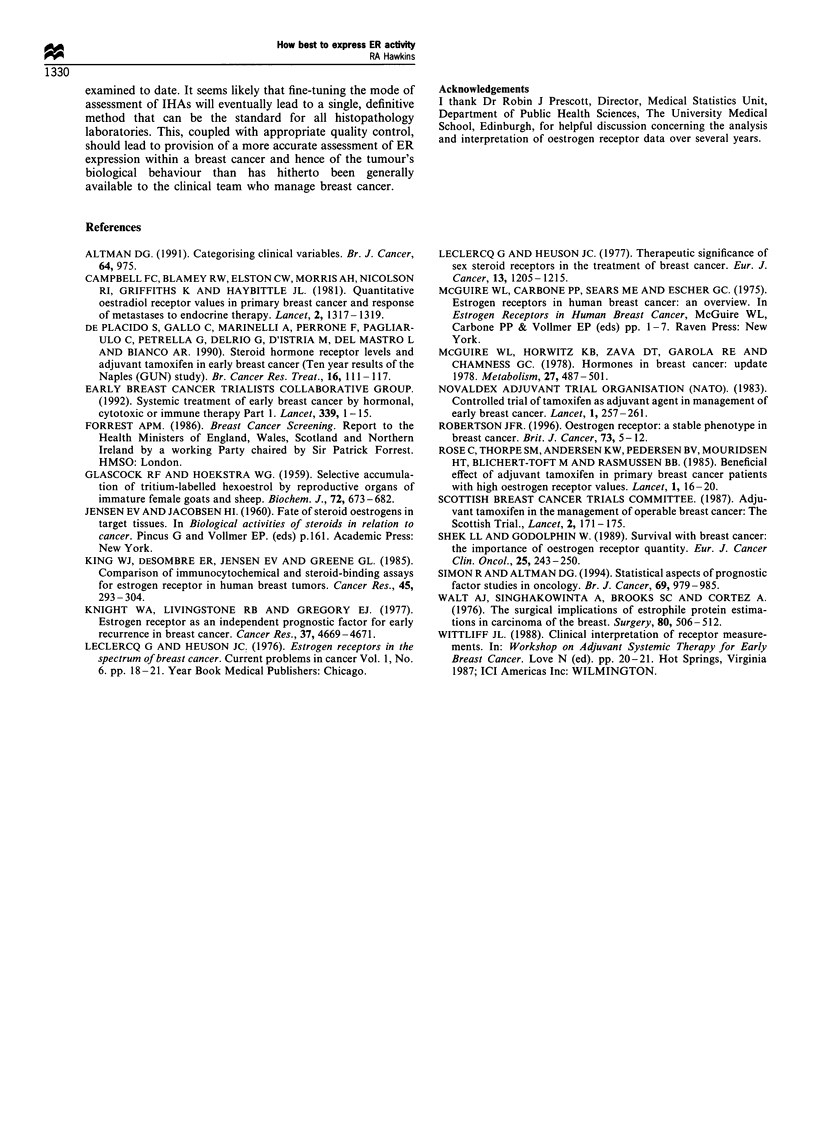# How best to express oestrogen receptor activity.

**DOI:** 10.1038/bjc.1996.544

**Published:** 1996-11

**Authors:** R. A. Hawkins


British Journal of Cancer (1996) 74, 1329–1330
© 1996 Stockton Press All rights reserved 0007–0920/96 $12.00

EDITORIAL

### How best to express oestrogen receptor activity

RA Hawkins

*University Department of Surgery, Royal Infirmary of Edinburgh Trust, Lauriston Place, Edinburgh EH3 9YW, UK.*

The oestrogen receptor (ER), discovered around 1960 (Glascock and Hoekstra, 1959; Jensen and Jacobsen, 1960), is present in the majority of breast cancers (70–100%, depending on method). Biochemical measurements of ER activity have long been known to be of value for *predicting* the outcome of endocrine therapy (NIH Consensus Meeting 1974, reported by McGuire *et al*., 1975) and also as a guide to *prognosis* (Walt *et al*., 1976; Knight *et al*., 1977). Since 1980, however, the use and acceptance of receptor assays in the management of breast cancer has had a chequered history for a variety of reasons, including: poor quality control of biochemical assays; poor control of specimen quality; changes in methodology; use of receptor measurements as a discontinuous variable (Altman, 1991; Simon and Altman, 1994) with an arbitrary 'cut-off' to decide receptor 'positivity/negativity'; the advent of the relatively non-toxic endocrine agent, tamoxifen; initial reports that benefit from adjuvant therapy with tamoxifen was unrelated to receptor activity (Novaldex Adjuvant Trial Organisation, 1983), although subsequent reports have disagreed (Rose *et al*., 1985; Scottish Breast Trials Committee, 1987; De Placido *et al*., 1990; Early Breast Cancer Trialists, 1992); the need for an adequate tumour specimen (50–250 mg); the advent of the Breast Cancer Screening Program (Forrest, 1986), which yields many impalpable tumours too small for biochemical assay; the widely quoted view that '8–10%' 'receptor-negative' tumours respond to endocrine therapy-likely to be untrue for the reasons discussed by, for example, Wittliffe, (1988), and Robertson, (1996); the misconception that tumour grade provides exactly the same information as oestrogen receptor measurements, but at a lesser cost.

Despite these difficulties, today in 1996, ER measurements are still recognised as being of importance for the management of breast cancer, and the advent (King *et al*., 1985) of immunohistochemical staining assays (IHAs) has afforded the opportunity for considerable improvements in the provision of receptor information as detailed below.

Now *all* tumours, irrespective of size, can be assayed – if need be retrospectively – on paraffin blocks.

The contribution to receptor assay measurements by contaminating benign tissue can be avoided.

The cost is lower (biochemical measurements are approximately £50 per specimen, IHA approx £3–10).

There is one problem, however, with the newer IHAs: quantitation of receptor activity is more difficult. Over the years, several studies have shown that receptor activity is a *continuous* variable and increasing *quantity* of receptor is associated with increasing probability of response/better prognosis (eg. Leclercq and Heuson, 1976, 1977; McGuire *et al*., 1978; Campbell *et al*., 1981; Shek and Godolphin, 1989).

In the present issue, Dr Diana Barnes and her colleagues thus address an issue of some importance. In this study, Dr Barnes' group has examined the relative merits of six different modes of expressing the results of IHA for ER in the paraffin sections from 170 patients treated with tamoxifen for metastatic breast cancer. The IHA results are assessed in relation to both clinical outcome and the results of a biochemical (ligand-binding) assay, carried out some 17 years previously. The patients studied were a selected, mixed group of 133 with operable primary disease, 23 with locally advanced disease and 14 with distant metastases at presentation, but overall showed a 51% rate of response.

In gauging the relative merits of the various modes of assessing IHA and the results of the biochemical assay, the ICRF group has expressed 'receptor status' by dividing the scores or values according to fixed criteria, usually defined previously by the originator of the mode examined. Thus 'receptor status' is expressed in a number of categories: two (biochemical assay, 'histo score'), three ('quick score') or four ('category score'). The key finding of the study is that, in general, *all* the modes for expressing IHA results were significantly related to outcome on endocrine therapy, with the 'quick score' of marginally greater significance. The biochemical ER assay, too, was significantly related to outcome but, as the authors note, it is unfair to draw conclusions concerning the comparison of such assays performed 17 years previously by the older ligand-binding assay (compare the more quantitative ER-EIA or enzyme immunoassay) with immunocytochemical assays in a *non*-prospective study.

ER expression is a continuous variable, with concentrations scattered over a large spectrum (Leclercq and Heuson, 1976, 1977). In Dr Barnes' paper, and in the real world, where IHAs now prevail for the reasons discussed above, it is probably necessary to down-grade the continuum into categories, for convenience. Nevertheless, the ER expressed as a continuum is the most prognostic/predictive form of the variable, measuring the probability of the tumour's biological behaviour. For this reason, modes of expressing ER results in only two categories are less informative and less predictive than those dividing into three or four categories, and the $\chi^2$ values and significances for the relationship between 'ER' and time to progression increase with increasing number of categories into which the 'ER-IHA' score is divided. In the light of this, it would seem imprudent to return to Jensen's original proposal of a two-category system, ie. 'ER-positive' and 'ER-negative', using an arbitrary cut-off. I believe that it is perfectly possible for the results of IHAs to be divided into a minimum of four categories ('negative', 'low', 'medium' and 'high') to yield prognostic/predictive information virtually equivalent to that provided, as a continuum, by a sensitive, quantitative biochemical assay such as the ER-EIA.

For the present, Dr Barnes and her colleagues have clearly demonstrated the value of ER-IHAs for predicting response to endocrine therapy and that all the modes of expressing the IHA score studied were of value. Whether the optimal mode of assessing IHAs has yet been reached remains to be seen. As ER concentrations are logarithmically distributed and the probability of response is sigmoidally related to the log of ER concentration (Leclercq and Heuson, 1976, 1977), it might be, for example, that division of IHA into four categories of doubling scores would prove more useful than the modes

Received 6 June 1996; accepted 10 June 1996

examined to date. It seems likely that fine-tuning the mode of assessment of IHAs will eventually lead to a single, definitive method that can be the standard for all histopathology laboratories. This, coupled with appropriate quality control, should lead to provision of a more accurate assessment of ER expression within a breast cancer and hence of the tumour's biological behaviour than has hitherto been generally available to the clinical team who manage breast cancer.

**Acknowledgements**
I thank Dr Robin J Prescott, Director, Medical Statistics Unit, Department of Public Health Sciences, The University Medical School, Edinburgh, for helpful discussion concerning the analysis and interpretation of oestrogen receptor data over several years.

### References

ALTMAN DG. (1991). Categorising clinical variables. *Br. J. Cancer*, **64**, 975.

CAMPBELL FC, BLAMEY RW, ELSTON CW, MORRIS AH, NICOLSON RI, GRIFFITHS K AND HAYBITTLE JL. (1981). Quantitative oestradiol receptor values in primary breast cancer and response of metastases to endocrine therapy. *Lancet*, **2**, 1317–1319.

DE PLACIDO S, GALLO C, MARINELLI A, PERRONE F, PAGLIARULO C, PETRELLA G, DELRIO G, D'ISTRIA M, DEL MASTRO L AND BIANCO AR. 1990). Steroid hormone receptor levels and adjuvant tamoxifen in early breast cancer (Ten year results of the Naples (GUN) study). *Br. Cancer Res. Treat.*, **16**, 111–117.

EARLY BREAST CANCER TRIALISTS COLLABORATIVE GROUP. (1992). Systemic treatment of early breast cancer by hormonal, cytotoxic or immune therapy Part 1. *Lancet*, **339**, 1–15.

FORREST APM. (1986). *Breast Cancer Screening*. Report to the Health Ministers of England, Wales, Scotland and Northern Ireland by a working Party chaired by Sir Patrick Forrest. HMSO: London.

GLASCOCK RF AND HOEKSTRA WG. (1959). Selective accumulation of tritium-labelled hexoestrol by reproductive organs of immature female goats and sheep. *Biochem. J.*, **72**, 673–682.

JENSEN EV AND JACOBSEN HI. (1960). Fate of steroid oestrogens in target tissues. In *Biological activities of steroids in relation to cancer*. Pincus G and Vollmer EP. (eds) p.161. Academic Press: New York.

KING WJ, DESOMBRE ER, JENSEN EV AND GREENE GL. (1985). Comparison of immunocytochemical and steroid-binding assays for estrogen receptor in human breast tumors. *Cancer Res.*, **45**, 293–304.

KNIGHT WA, LIVINGSTONE RB AND GREGORY EJ. (1977). Estrogen receptor as an independent prognostic factor for early recurrence in breast cancer. *Cancer Res.*, **37**, 4669–4671.

LECLERCQ G AND HEUSON JC. (1976). *Estrogen receptors in the spectrum of breast cancer*. Current problems in cancer Vol. 1, No. 6. pp. 18–21. Year Book Medical Publishers: Chicago.

LECLERCQ G AND HEUSON JC. (1977). Therapeutic significance of sex steroid receptors in the treatment of breast cancer. *Eur. J. Cancer*, **13**, 1205–1215.

MCGUIRE WL, CARBONE PP, SEARS ME AND ESCHER GC. (1975). Estrogen receptors in human breast cancer: an overview. In *Estrogen Receptors in Human Breast Cancer*, McGuire WL, Carbone PP & Vollmer EP (eds) pp. 1–7. Raven Press: New York.

MCGUIRE WL, HORWITZ KB, ZAVA DT, GAROLA RE AND CHAMNESS GC. (1978). Hormones in breast cancer: update 1978. *Metabolism*, **27**, 487–501.

NOVALDEX ADJUVANT TRIAL ORGANISATION (NATO). (1983). Controlled trial of tamoxifen as adjuvant agent in management of early breast cancer. *Lancet*, **1**, 257–261.

ROBERTSON JFR. (1996). Oestrogen receptor: a stable phenotype in breast cancer. *Brit. J. Cancer*, **73**, 5–12.

ROSE C, THORPE SM, ANDERSEN KW, PEDERSEN BV, MOURIDSEN HT, BLICHERT-TOFT M AND RASMUSSEN BB. (1985). Beneficial effect of adjuvant tamoxifen in primary breast cancer patients with high oestrogen receptor values. *Lancet*, **1**, 16–20.

SCOTTISH BREAST CANCER TRIALS COMMITTEE. (1987). Adjuvant tamoxifen in the management of operable breast cancer: The Scottish Trial., *Lancet*, **2**, 171–175.

SHEK LL AND GODOLPHIN W. (1989). Survival with breast cancer: the importance of oestrogen receptor quantity. *Eur. J. Cancer Clin. Oncol.*, **25**, 243–250.

SIMON R AND ALTMAN DG. (1994). Statistical aspects of prognostic factor studies in oncology. *Br. J. Cancer*, **69**, 979–985.

WALT AJ, SINGHAKOWINTA A, BROOKS SC AND CORTEZ A. (1976). The surgical implications of estrophile protein estimations in carcinoma of the breast. *Surgery*, **80**, 506–512.

WITTLIFF JL. (1988). Clinical interpretation of receptor measurements. In: *Workshop on Adjuvant Systemic Therapy for Early Breast Cancer*. Love N (ed). pp. 20–21. Hot Springs, Virginia 1987; ICI Americas Inc: WILMINGTON.